# Investigating Tungsten Carbide Micro-Hole Drilling Characteristics by Desktop Micro-ECM with NaOH Solution

**DOI:** 10.3390/mi9100512

**Published:** 2018-10-11

**Authors:** Yung-Yi Wu, Dong-Yea Sheu

**Affiliations:** 1 Graduate Institute of Mechanical & Electrical Engineering, CMEE, National Taipei University of Technology, Taipei 10608, Taiwan; 2 Graduate Institute of Manufacturing Technology, National Taipei University of Technology, Taipei 10608, Taiwan; dongyea@ntut.edu.tw

**Keywords:** tungsten carbide WC, micro-hole, desktop micro-ECM

## Abstract

Due to their hardness and low tool wear, tungsten carbides are widely used in industrial applications, such as spray nozzles, wire drawing dies and spinning nozzles. However, there is no conventional machining process that is capable of fabricating micro-holes, slots and complicated shapes in tungsten carbide. In this study, a low-cost desktop micro electro-chemical machining (ECM) was developed to investigate the characteristics of tungsten carbide micro-hole drilling. The performance parameters of the machining conditions by desktop micro-ECM, such as the machining time, material removal rate, relative tool wear rate, surface quality and dimensional accuracy, were also investigated in this study. The experimental results demonstrate that the low-cost desktop micro-ECM could fabricate micro-holes in the tungsten cemented carbide (WC-Co) workpiece.

## 1. Introduction

Tungsten carbide (WC) is a material that is widely used by military–industrial complexes and in multiple important fields, including metallurgy and aerospace, due to its excellent physical and chemical properties [1,2]. Pure WC is very brittle. However, if it is bound with a small amount of titanium, cobalt or other metals by sintering, the WC brittleness can be reduced. The interaction between the Co-based binders and WC grains in the early stages of liquid-phase sintering can be strongly affected by the carbon content of the binders [3]. Tungsten cemented carbide (WC-Co) has a series of excellent properties, such as hardness, strength, toughness, wear resistance and corrosion resistance. The most important of these are its high hardness and wear resistance. Tungsten cemented carbide can also be used to produce rock drilling tools, metal grinding tools, precision bearings and nozzles etc.

WC-Co meets the requirements of the hardness and lower wear for micro-nozzle applications. Grinding, laser beam machining (LBM), electron beam machining (EBM), electrical discharge machining (EDM) and electro-chemical machining (ECM) processes are popular methods for machining WC-Co materials [4]. Conventional grinding is also able to create a high-quality surface on the workpiece, but it is still difficult to manufacture a complicated shaped product and the material removal rate is low. Micro-EDM and Micro-ECM are popular for drilling micro-holes into a WC-Co workpiece [5,6].

Furthermore, some machining processes, such as micro-mechanical drilling, LBM and EBM, are available for the mass or semi-mass production of micro-hole fabrication. The micro-EDM process is still an excellent method for micro-hole fabrication since the development of wire electro-discharge grinding (WEDG) technology [7]. However, the wear of the micro-tools is the primary critical problem for the EDM process. However, considering that the product is burr-free and lacks the limitation of hardness, ECM is possibly a new process for tungsten carbide machining [8]. ECM is also called electrolytic machining, which belongs to the class of nonconventional machining methods. This results in more advantages. For example, all metal materials can be machined no matter how hard the material is. The cathode tool will not break during the machining process. After machining, the workpiece will not have any residual stress remaining on its surface. Finally, the machining reproducibility of ECM is better than EDM [9].

ECM has other advantages, which include: No mechanical stress impact; no thermal impact resulting in high surface quality; independence of material hardness and brittleness; and no tool wear [10]. Electrochemical machining uses the “anode” (positive voltage) as the “workpiece” and uses the “cathode” (negative voltage) as the “tool electrode.” The anode and cathode of the workpiece and the tool electrode were created by oxidation and reduction in the electrolytes. ECM is a difficult-to-control process due to stray corrosion [11]. In ECM, both the tool electrode and the workpiece are submerged in an electrically conductive electrolyte, which is usually an aqueous solution. This was chosen to be sodium hydroxide (NaOH) in this study. A direct current (DC) potential is applied between the two electrodes, ensuring that the workpiece becomes the anode [12]. The applied potential causes a current to flow between the electrodes, dissolving the anode material in the process. The parameters of ECM, such as the machining time and electrolytes, for the WC-Co workpiece micro-hole drilling were investigated in this paper.

## 2. Materials and Methods

**Scheme 1:**
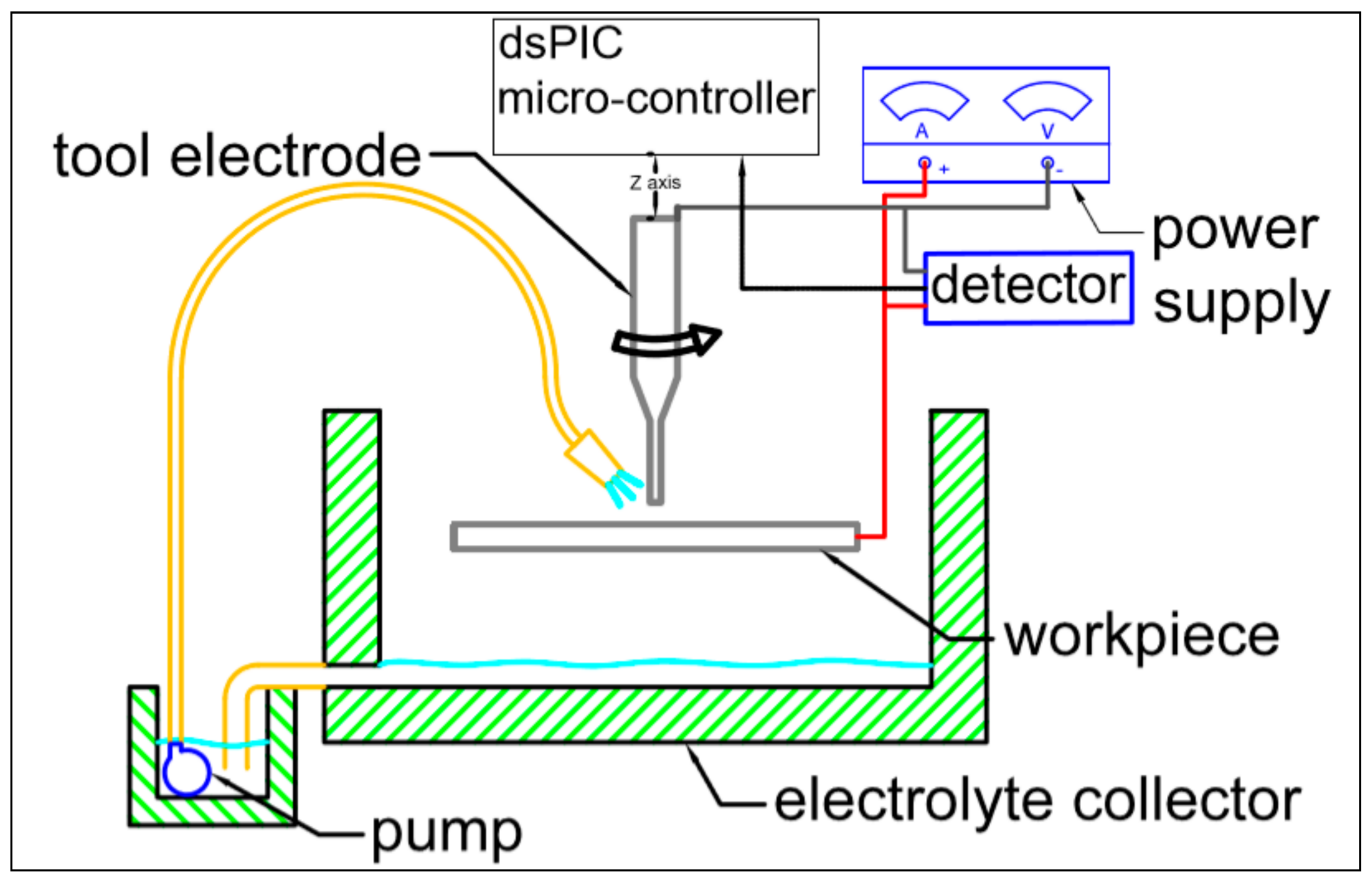
Schematic illustration of the desktop micro electro-chemical machining (ECM) system.

**Figure 1:**
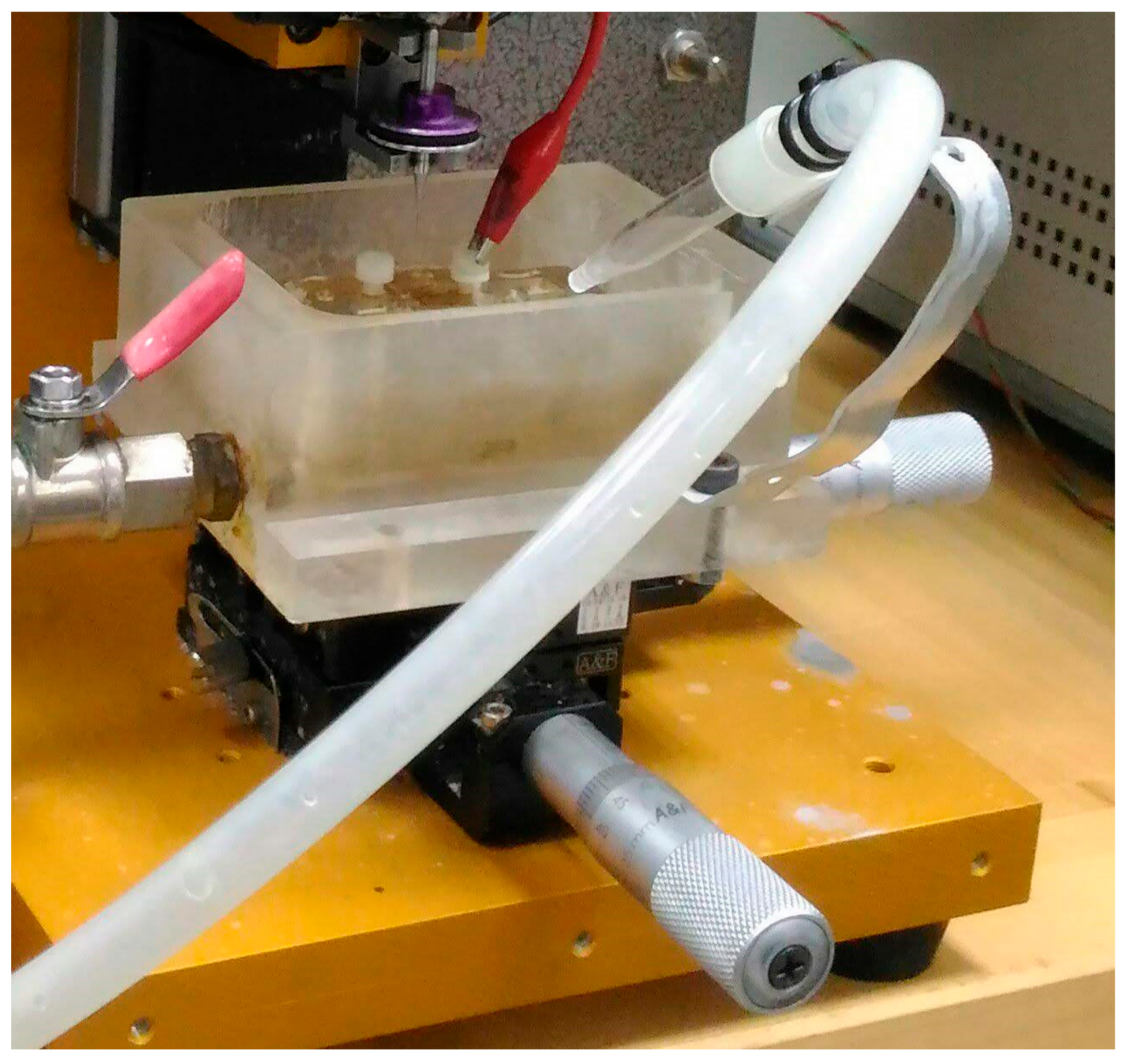
The X–Y table and electrolyte collector of the desktop micro-ECM.

**Figure 2:**
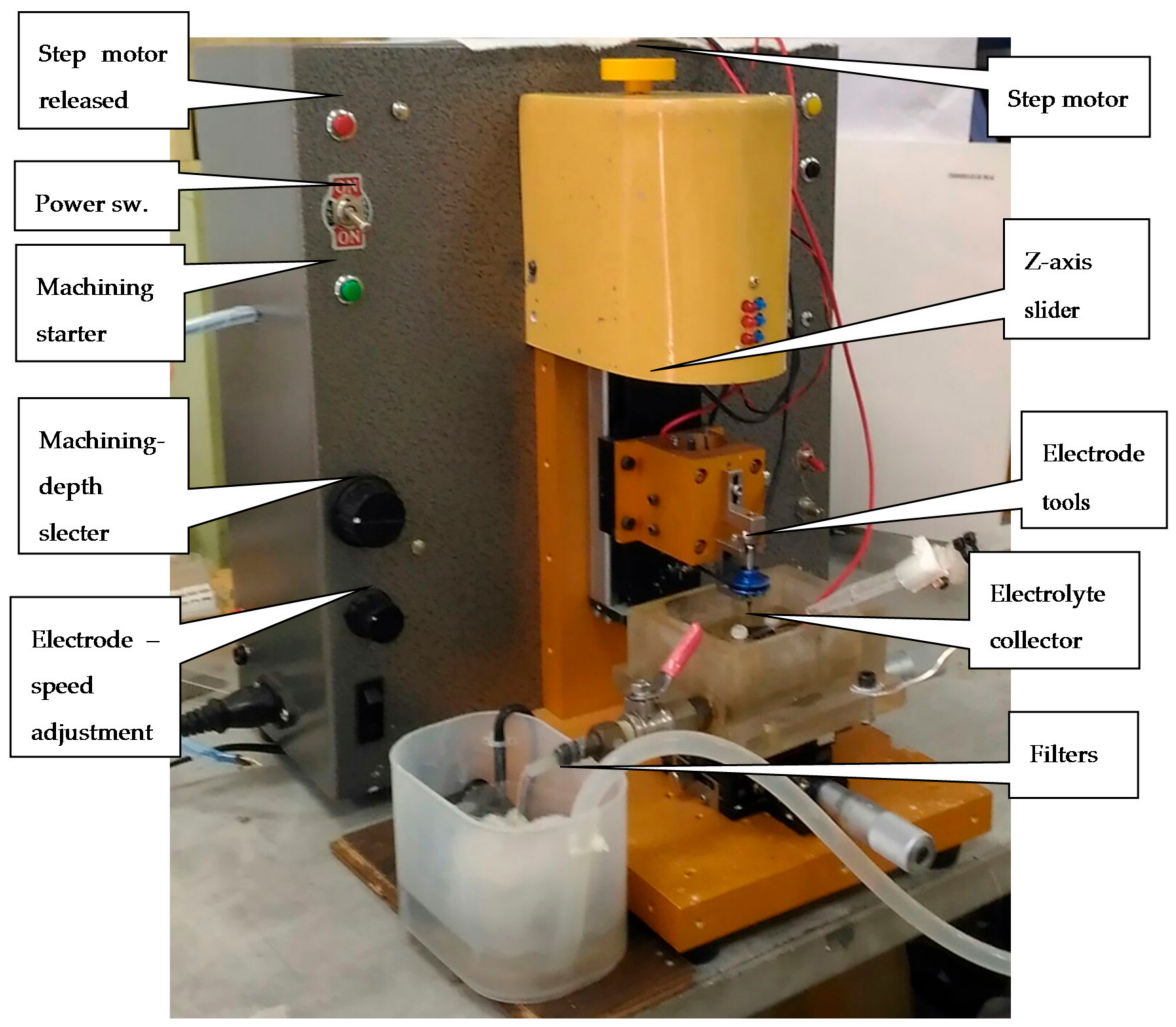
Entire structure of the desktop micro-ECM.

For the experimental investigations, a desktop micro-ECM system was developed. A schematic illustration of the desktop micro-ECM and its basic components, as well as their interactions, are shown in Scheme 1. The desktop micro-ECM, which is shown in Figure 1, has *X*, *Y* and *Z* axes for micro-hole drilling. The X–Y direction is manually controlled by the micro-meter screw gauge and the *Z*-axis can be controlled by the microcontroller unit (MCU). The most important parts of the desktop micro-ECM are the V-shaped block because the function of the V-shape is to maintain the concentricity and straighten the spindle electrode tools. The spindle tools are mounted on the V-shaped block and rotated by a DC motor. The electrolyte collector contains the spindle tools and electrolyte flow. The rotation speed is changeable. The linear straightness and roundness are important for ensuring the high accuracy of micro-hole drilling. The entire structure of the desktop micro-ECM is shown in Figure 2.

**Figure 3:**
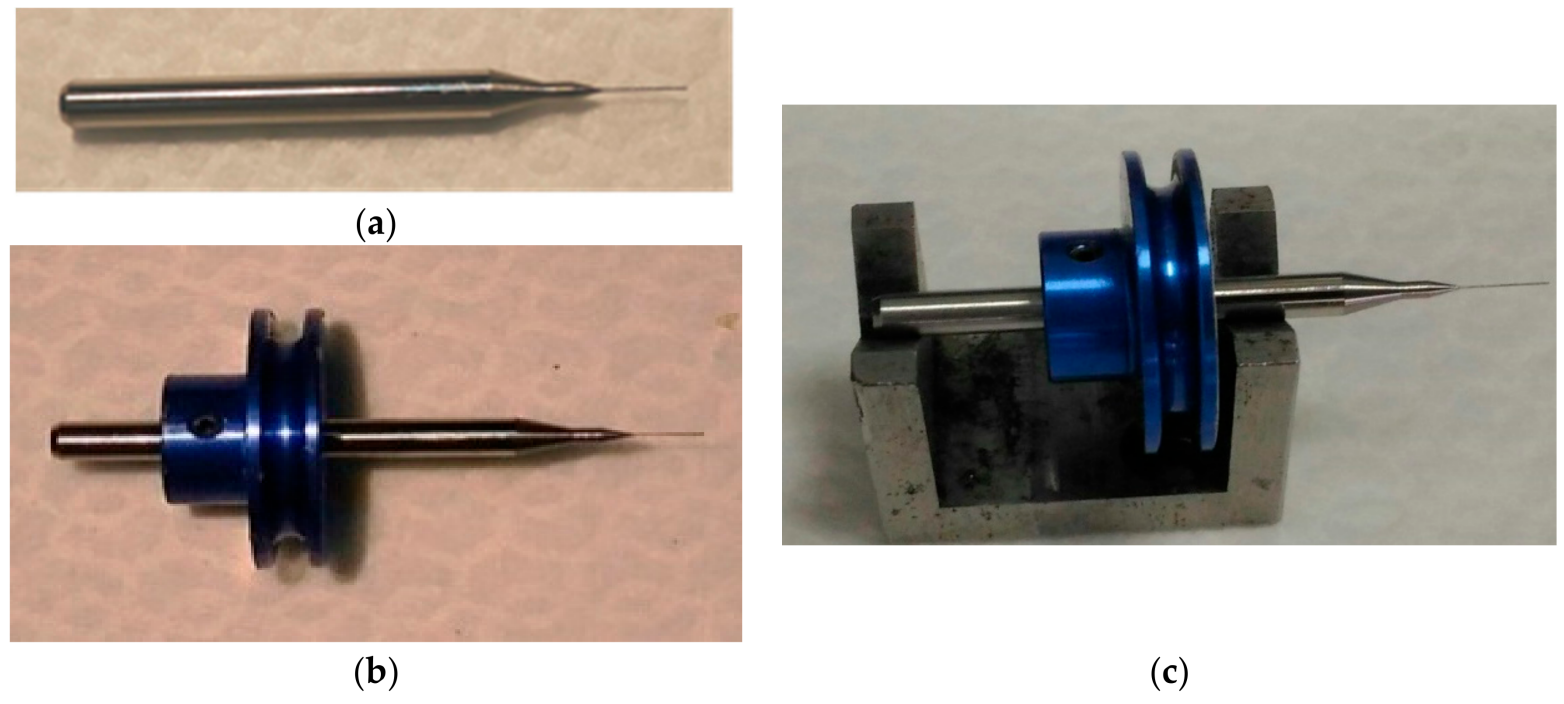
The spindle micro-tool rotates on the V-shaped block with pulley. (**a**) Spindle electrode tool. (**b**) Spindle micro-tool with pulley. (**c**) Micro-tool rotates on the V-shaped block.

The desktop micro-ECM uses the spindle electrode tools from the components found in the market, which can be fabricated to have a tolerance of approximately 3 μm in diameter and length. The material used for the tool is tungsten cemented carbide, in which the diameter of the shank is 0.3 mm, while the diameter and the length of the drill are 150 μm and 5 mm, respectively. The pulley was mounted onto the shank directly and the spindle micro-tool rotates on the V-shaped block, which is shown in Figure 3. The accuracy of the rotation roundness could be maintained below 0.5 μm. The top and bottom of the V-shaped block will maintain the spindle electrode tools centrally and keep it stable without any vibration. Although the WC has a lower tool wear rate for micro-ECM, this WC is not available for mass production by the grinding process due to its insufficient toughness. The WC-Co material can be mass-produced by the mechanical grinding process. However, in the commercial market, it is still difficult to produce the spindle tools with a diameter less than 0.05 mm due to the mechanical grinding force. With WEDG, it is possible to fabricate spindle tools with a diameter of 50 μm. The diameter of the micro-electrode tool could be adjusted by aligning the X-axis position by the micro-meter screw gauge. The aspect ratio is only 3 or 4 times. This indicates that the low aspect ratio of spindle tools is not available for mass micro-hole drilling by desktop micro-ECM.

**Figure 4:**
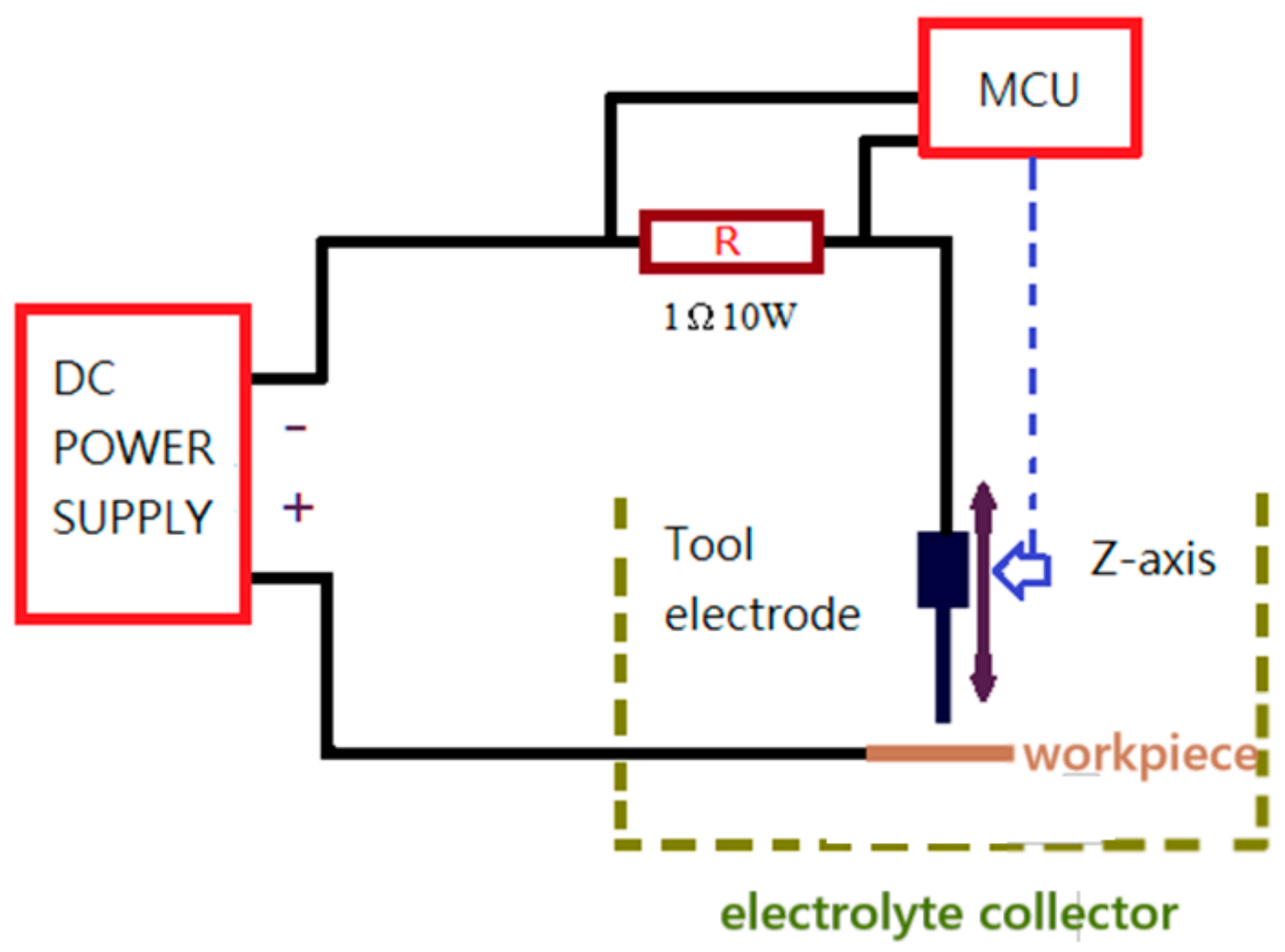
Detection of circuit.

**Figure 5:**
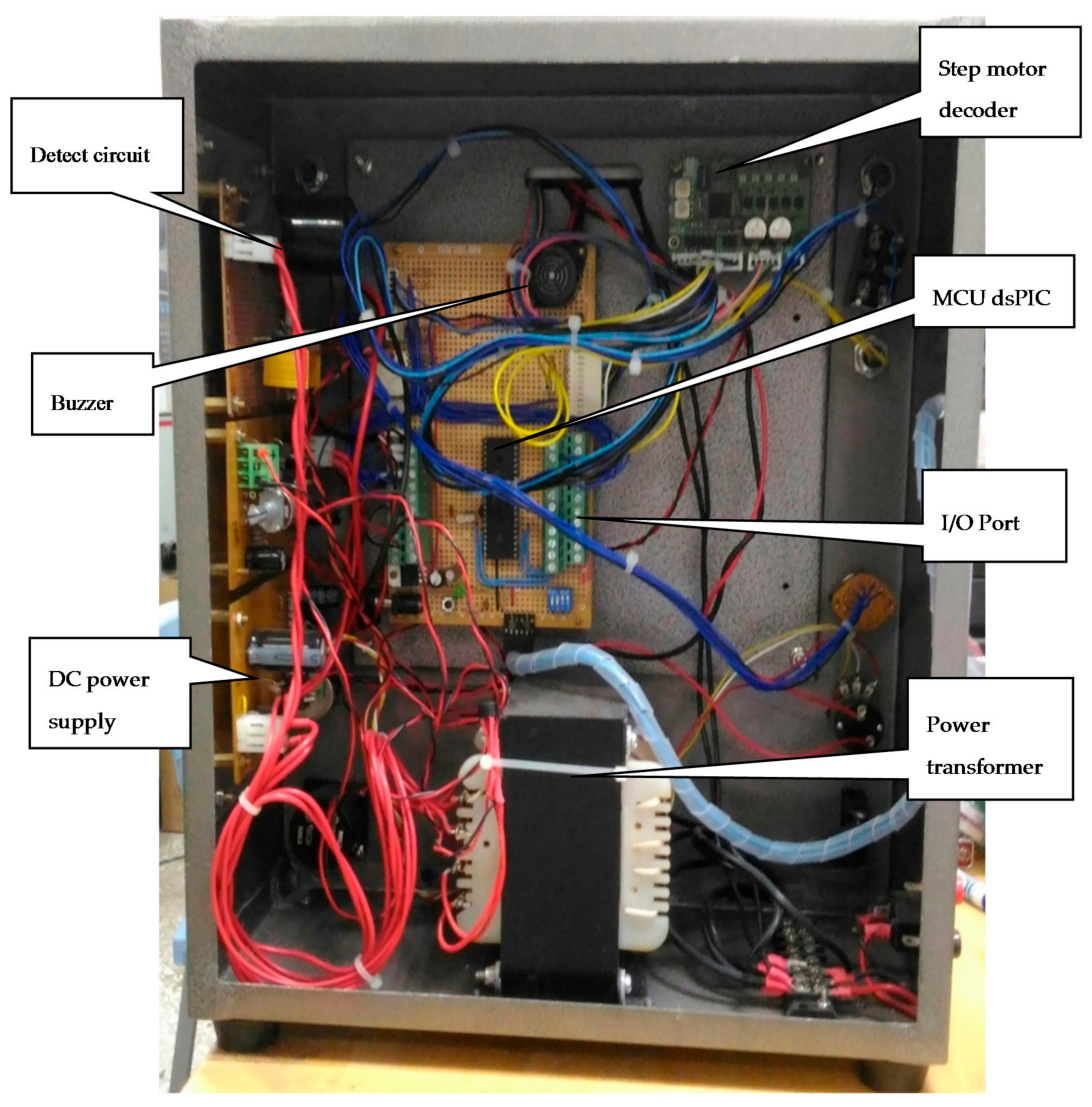
The internal structure of the micro-ECM.

A conventional desktop micro-ECM is operated by a NC controller. Both the position alignment and the scanning process with tool compensation is possible. However, the large cost of the conventional desktop micro-ECM makes the ECM process unpopular for micro-hole drilling. This present study used the dsPIC30F4011 of the microchip corp. (Chandler, AZ, USA) as the MCU to control the spindle tool motion on the *Z*-axis and to detect the electrolyte current of the circuit resistor and determine the feed rate, which is shown in Figure 4. The I/O port of the dsPIC connection provides the selection of the drilling depth. The desktop micro-ECM monitoring is based on the voltage and current feedback signals from the current sensors of analog-to-digital converters (ADC). Short circuit detection and prevention, thus enabling better process stability and precise machining, are further advantages that result from using the desktop micro-ECM to control the processes, with the internal structure of the device being shown in Figure 5. Most of the electrolyte current of ECM depends on the machining parameters, such as working voltage, pulse duty cycle and electrolyte concentration. The optimization of these parameters can be conducted in order to achieve machining with no spark effect and to reduce electrode wear. By only pushing the start button, the desktop micro-ECM can produce micro-holes automatically. The micro-tools and micro-hole fabrication could be performed on the same desktop micro-ECM.

## 3. Results and Discussion

### 3.1. Micro-Hole Drilling by Desktop Micro-ECM

**Figure 6:**
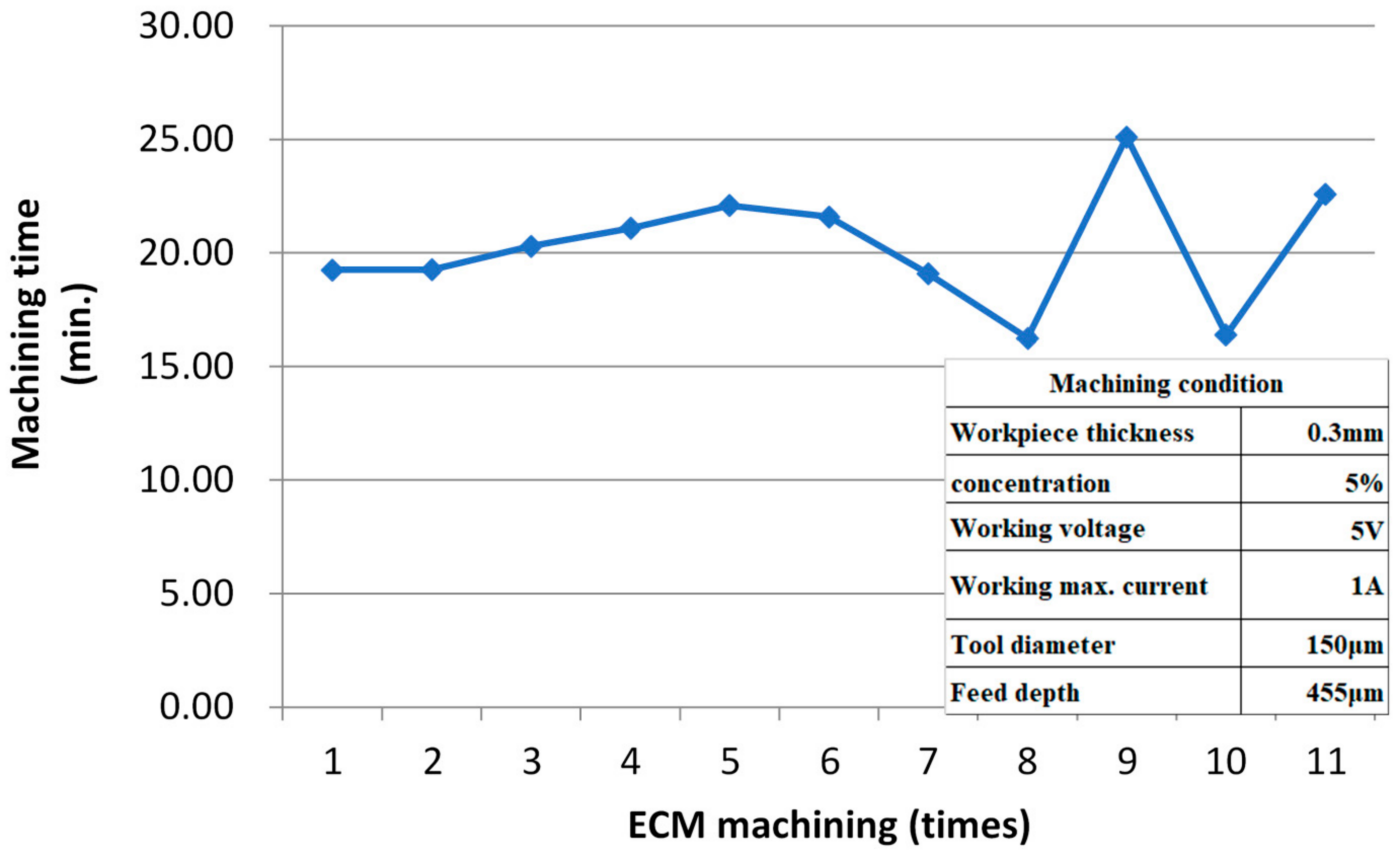
Micro-hole micro-ECM machining time.

**Figure 7:**
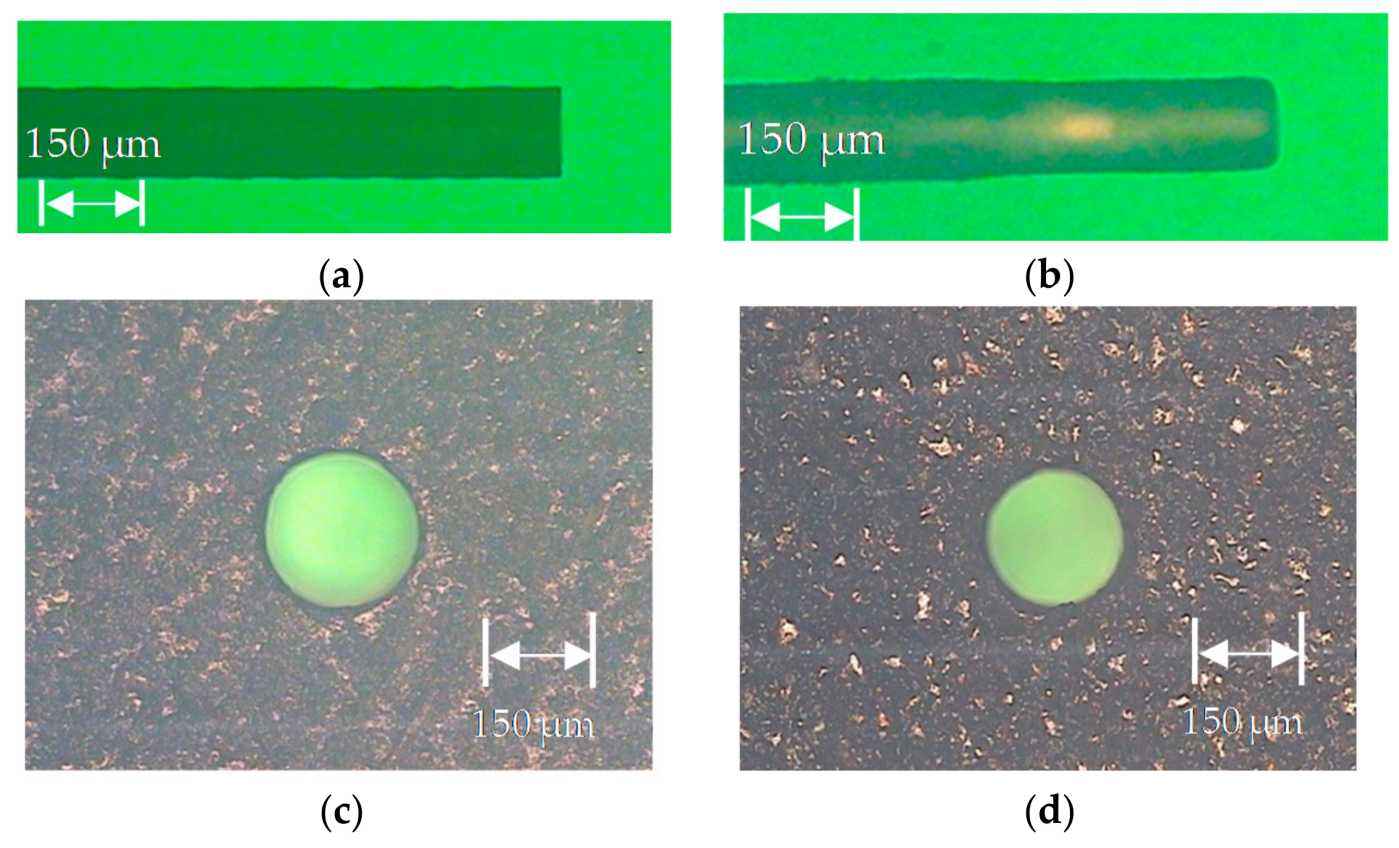
Different views of micro-tools and hole drilling by micro-ECM. (**a**) Before ECM drilling tool (/φ150 μm). (**b**) After ECM drilling tool (/φ135 μm). (**c**) Inlet (178 μm). (**d**) Outlet (147 μm).

In this present study, the diameter (150 μm) of the electrode tools on the micro-spindle was used for fabrication in order to achieve the mass production of the micro-hole drilling by micro-ECM. The machining parameters of the desktop micro-ECM is shown in Table 1, which uses a power voltage of 5–20 V, concentration of 5% sodium hydroxide (NaOH), diameter of 150 μm for the micro-tools and machining depth of 455 μm. Furthermore, the WC-Co had a workpiece thickness of 0.3 mm in order to avoid drilling the taper holes and to produce high-quality micro-holes, which means that the machining depth must exceed the workpiece thickness. From the micro-ECM drilling experiments, the machining time was about 20 min (Figure 6). After the experiment, the electrode of the tools and micro-holes were measured by the optical microscope MF-UN1010TH of Mitutoyo Corp (Kawasaki, Japan). The holes had a dissociation of 15 μm after drilling, while there was nearly no tool wear. Different views of the micro-holes of ECM drilling are shown in Figure 7. The micro-tool exhibits evidence of wear during the ECM process due to the dissociation generation.

### 3.2. Limitations of Machining Depth

**Figure 8:**
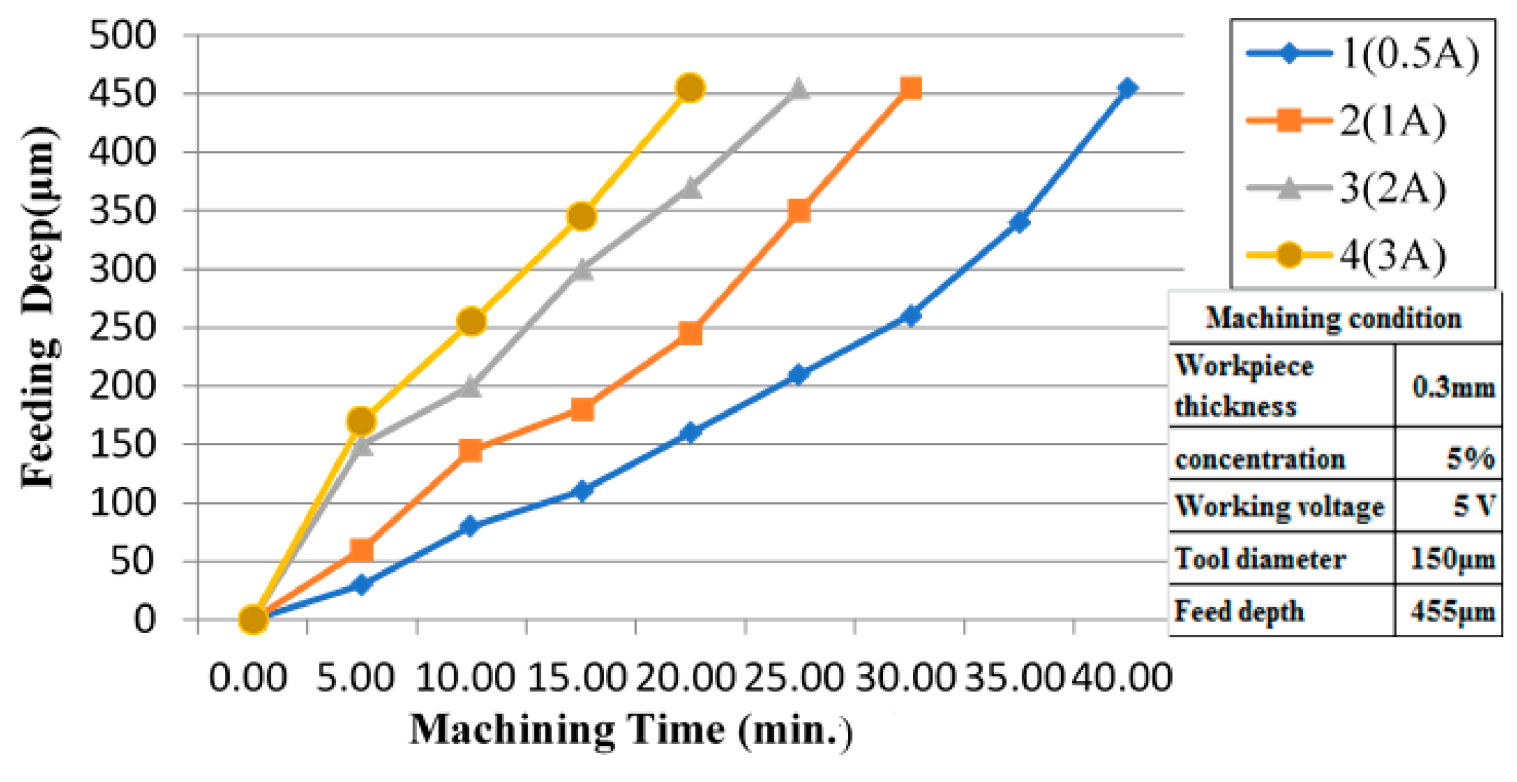
Feeding depth in different currents.

**Figure 9:**
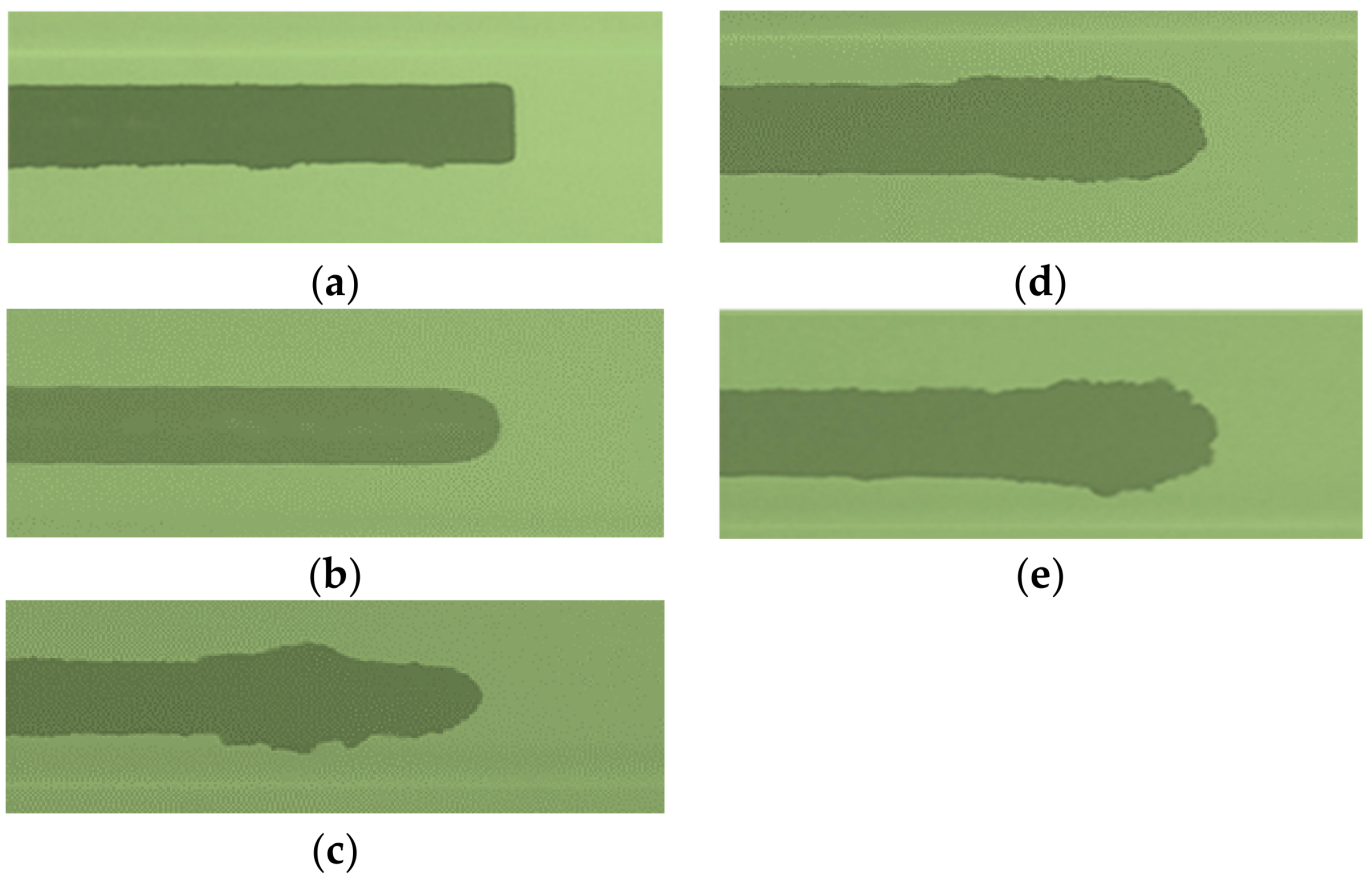
Micro-tool length changes in tungsten cemented carbide (WC-Co) by the desktop micro-ECM. (**a**) New tool with a diameter of 0.15 mm. (**b**) After machining in 5 V; tool length wear is 25 μm. (**c**) After machining in 10 V; tool length wear is 5 μm. (**d**) After machining in 15 V; tool length deposition is +12 μm. (**e**) After machining in 20 V; tool length deposition is +15 μm.

The micro-ECM hole drilling is dependent on the current density in the electrolytes. The sodium hydroxide (NaOH) electrolyte solution has a 5% concentration and the working voltage is 5 V. In order to compare the effect of the machining depth of ECM at different currents, we used a constant DC pulse working voltage in 5 V with a target value of 455 under the same machining conditions. The current level is calculated in increments every 5 min, data as shown in Table 2. It took about 48 min in 0.5 A, 34 min in 1 A, 27 min in 2 A and 23 min in 3 A. The feeding depth of the micro-tool during WC-Co ECM micro-hole drilling with different currents is shown in Figure 8. The machining efficiency increases with an increase in the current. The length of the micro-tools changes with the gap working voltage when using the sodium hydroxide (NaOH) electrolytic solution concentration of 10%, which is shown in Figure 9. The length of the tools increases due to the artefacts of deposition when the gap working voltage is larger than 10 V. It is clear that some remnants from deposition were attached to the tools, which is shown in Figure 9. The length of the micro-tools does not wear but increases due to the deposition of tungsten oxide. The non-linear electrolytic machining time and the tool electrode of the micro-ECM are changing. From the above experiments, we determined that when we maintain the sodium hydroxide (NaOH) electrolyte concentration at 10% with a maximum electrolytic current of 1 A, the electrode tool will produce some insulating materials as it deposited a layer of oxide onto the surface with a working voltage higher than 12 V. As expected, the tool will not deposit materials at a working voltage less than 12 V, which will affect the electrochemical processing. However, if we lower the working voltage, the processing time will be extended and this affects the quality of the electrochemical machining process.

### 3.3. Electrode Tool Wear and Roundness

**Figure 10:**
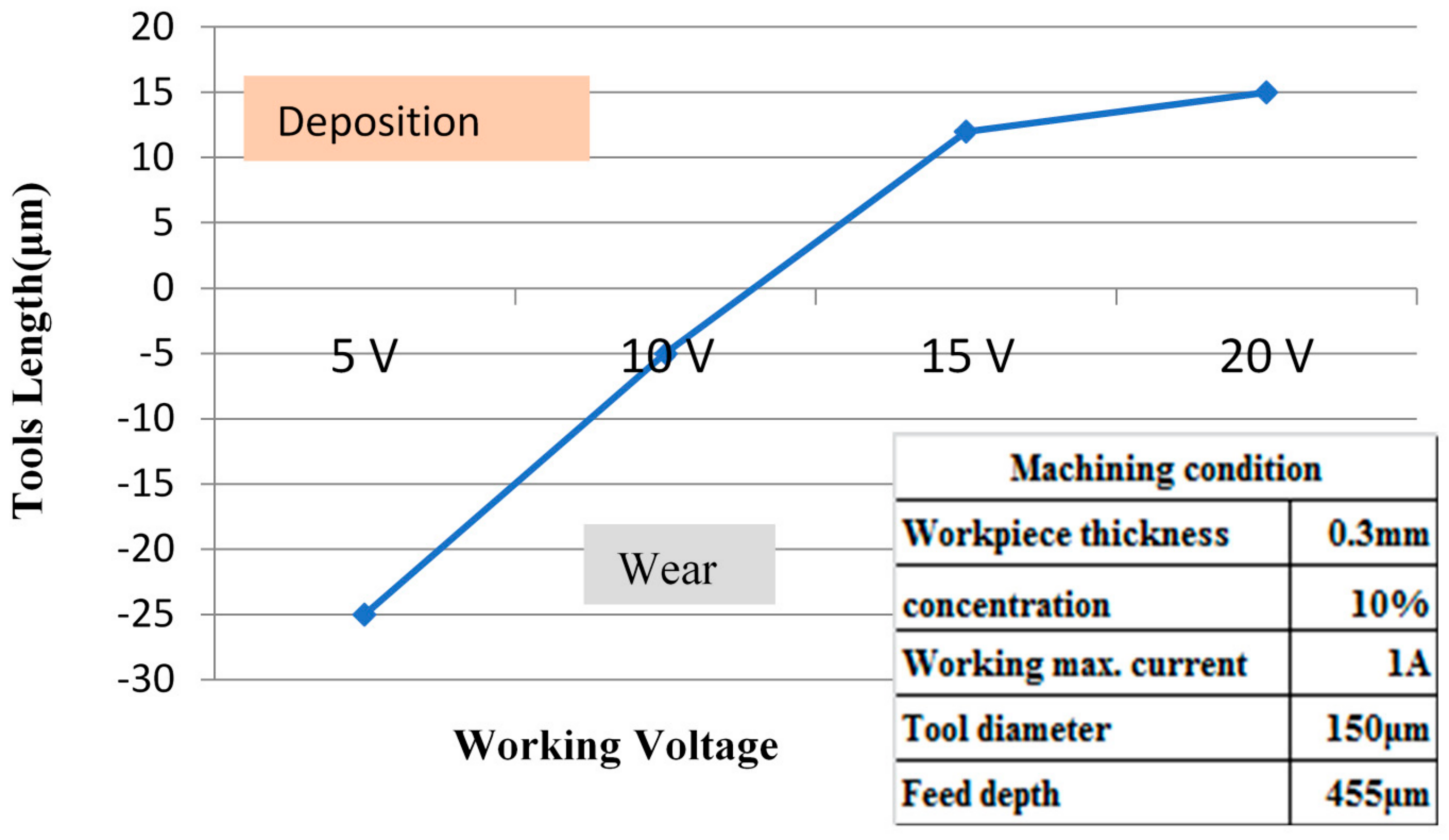
Micro-tool changes in WC-Co by the desktop micro-ECM.

**Figure 11:**
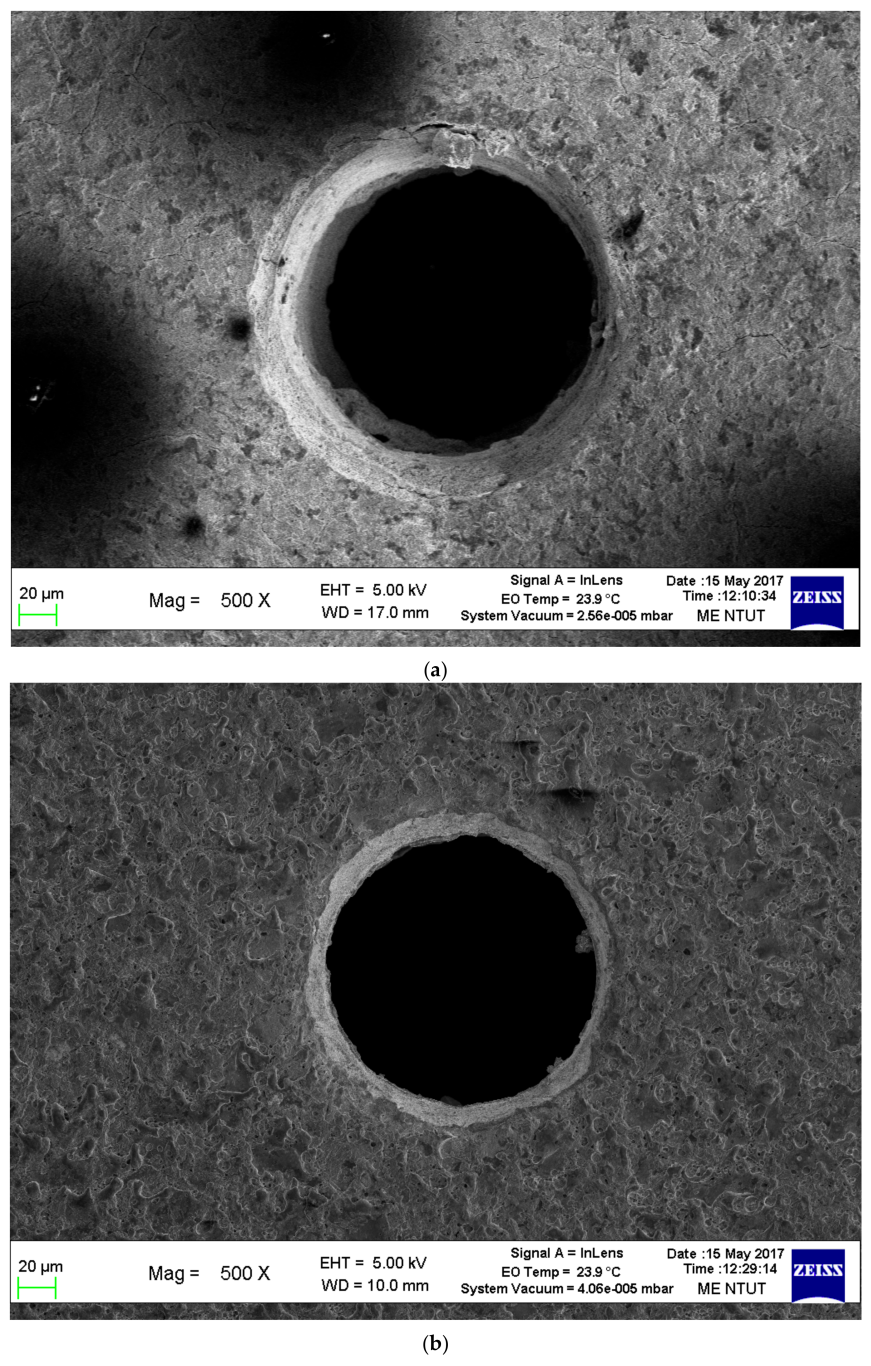

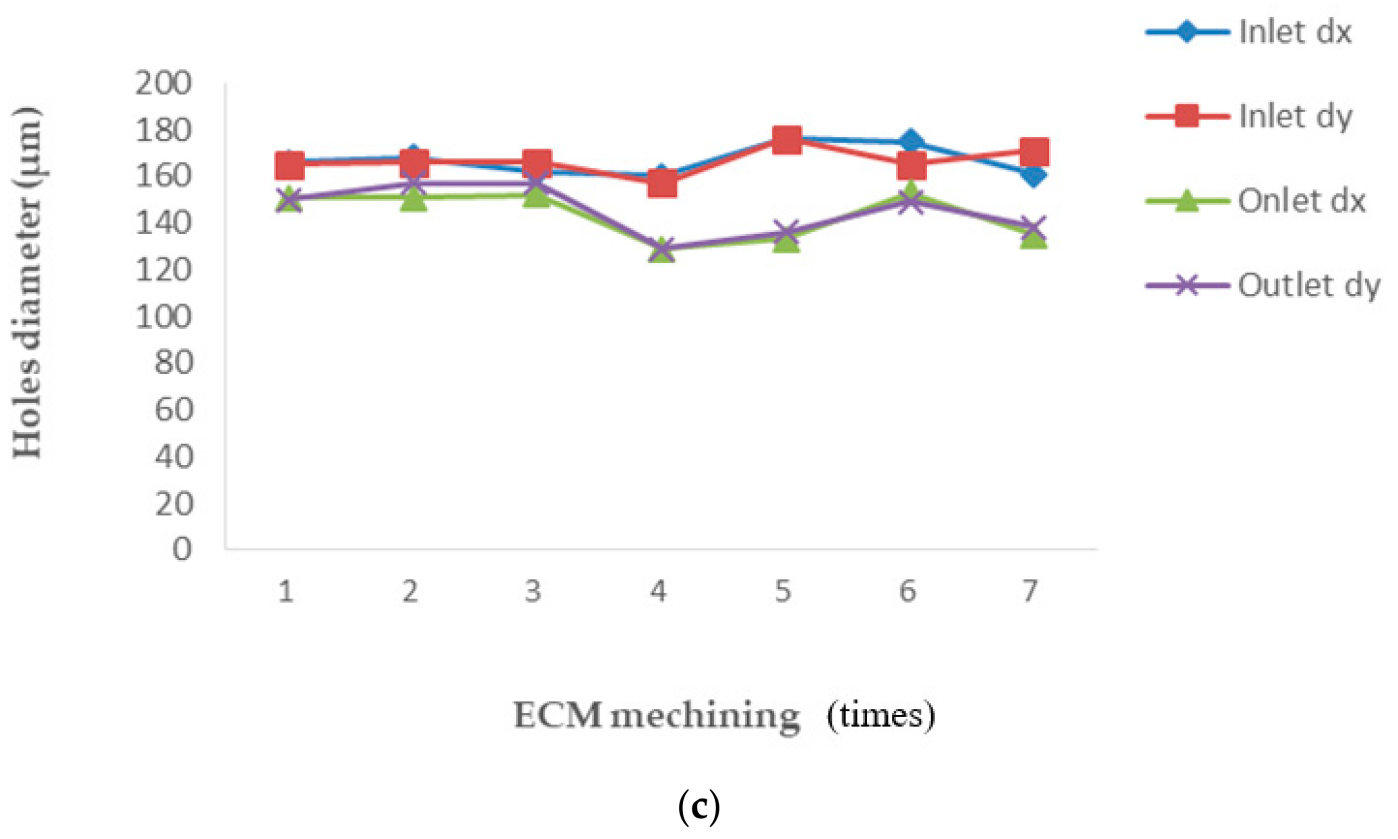
Observation inlet and outlet of Tungsten cemented carbide (WC-Co) micro-holes. (**a**) The inlet of WC-Co micro-holes by scanning electron microscope (SEM); (**b**) The outlet of WC-Co micro-holes by SEM. (**c**) Inlet and outlet micro-hole diameters for the *X* and *Y* axes.

**Figure 12:**
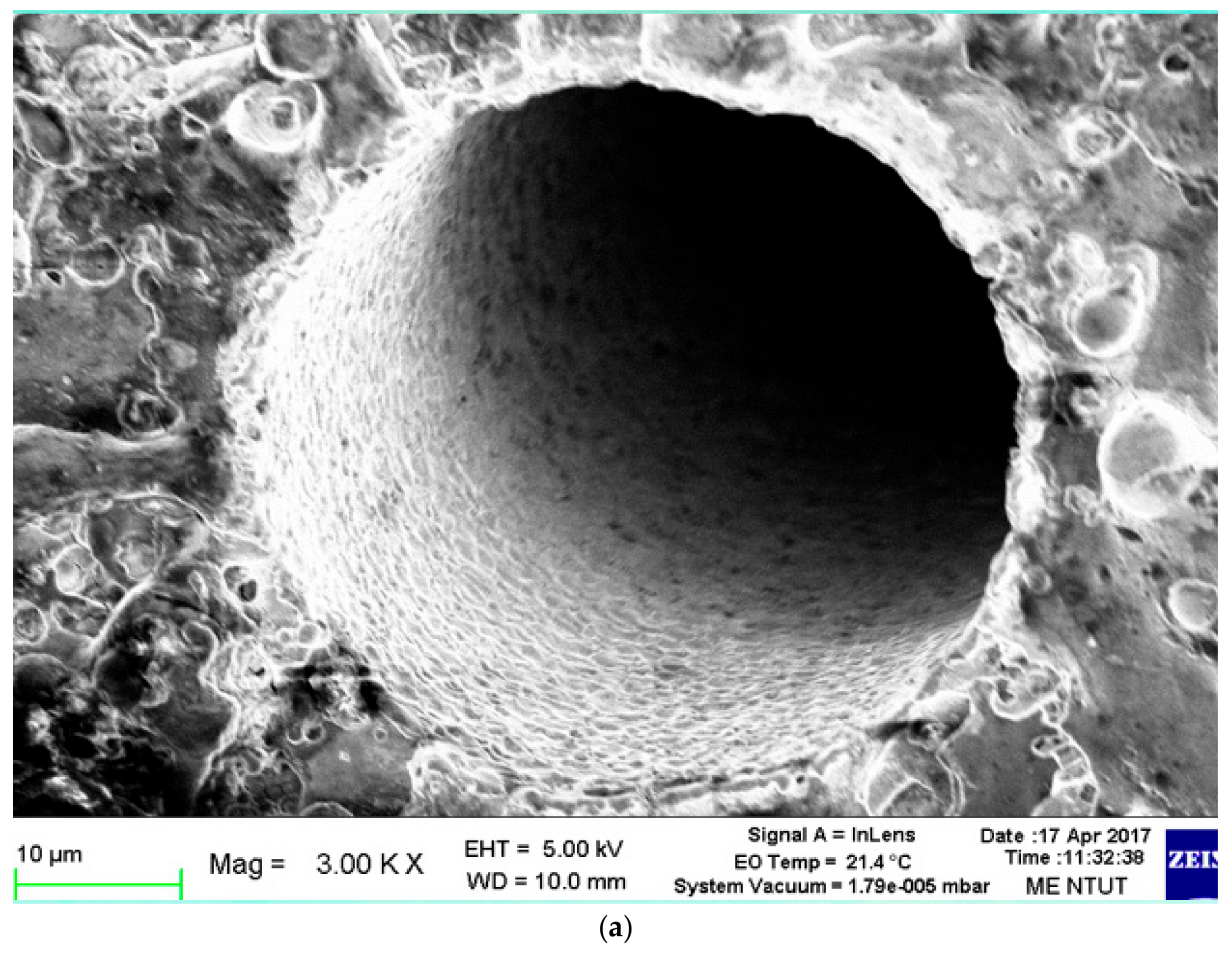

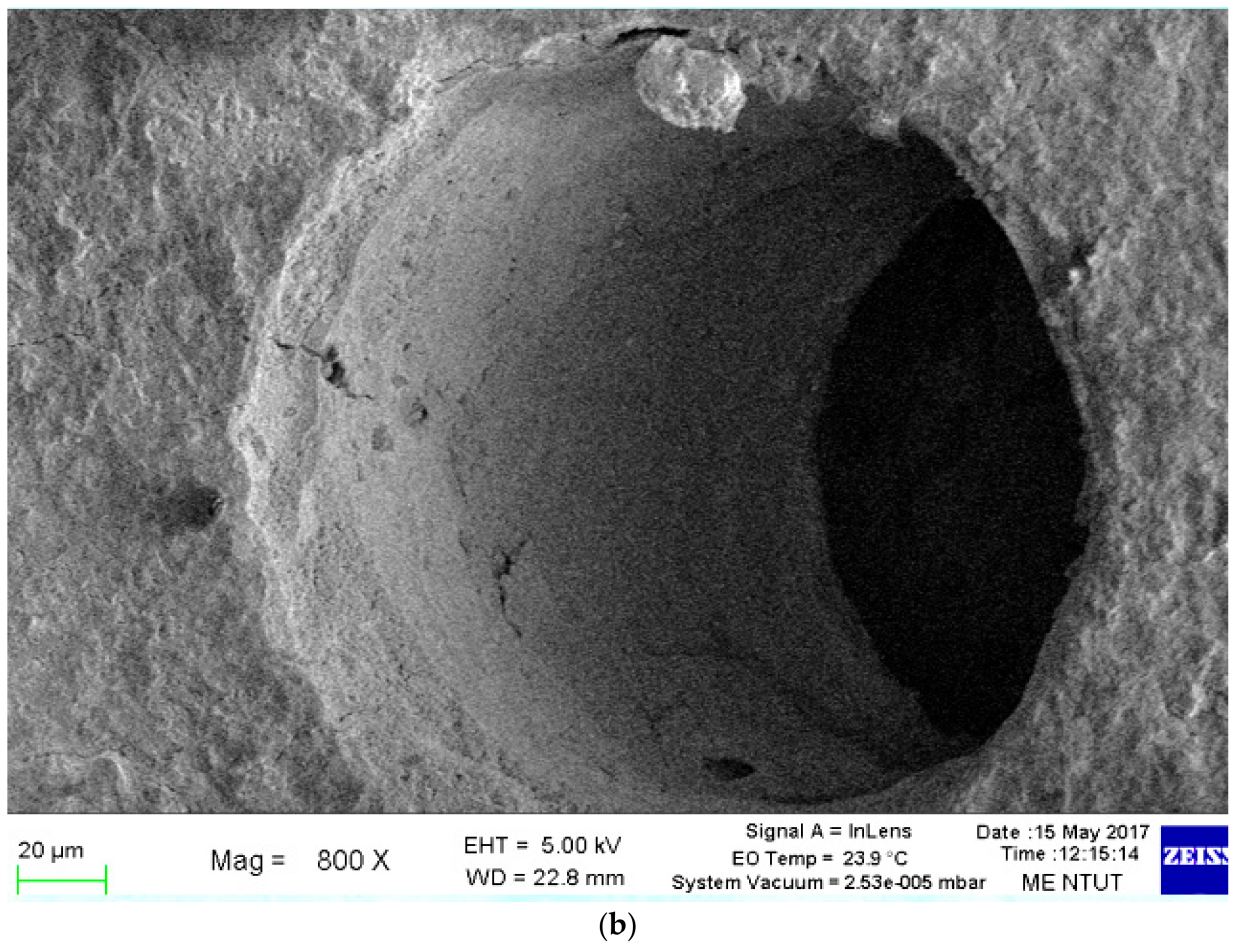
Micro electrical discharge machining (EDM) compared with the micro-ECM machining process. (**a**) The surface of EDM presented to more uniformed like a grapefruit peel. (**b**) The surface of ECM presented to be smooth and evenly circled.

In general, micro-tools should be free of tool wear in the electrolytic machining process. However, in this present study, when the voltage between the two electrodes is higher than 12 V, an insulating oxide will be deposited on the surface of the microelectrode tool, which makes the tool longer by about 15 μm at 20 V and about 12 μm at 15 V. When the gap working voltage is lower than 12 V, there will be no deposits attaching to the microelectrode tool. However, due to the difficulty in the feeding control of the ECM, a partial discharge will occur, causing tool wear. The wear of the tool is about 25 μm at 5 V and about 5 μm at 10 V. These micro-tool changes are shown in Figure 10 and data as shown in Table 3. The inlet and outlet of the micro-holes are shown in Figure 11a,b. The quality of surface roughness and roundness of the micro-holes can be observed from the measurements of the horizontal inlet diameter of 167 μm and the vertical inlet diameter of 167 μm as well as the horizontal outlet diameter of 143 μm and the vertical outlet diameter of 145 μm, which are shown in Figure 11c and data as shown in Table 4. This phenomenon indicates that there were some small electrical discharge sparks during micro-ECM erosion. Differing from ECM, EDM uses a higher voltage electric field to conduct spark discharge machining compared to the micro-ECM. By using scanning electron microscope (SEM) to scan the surface of the drilling holes of the micro-EDM and those of the micro-ECM machining process as shown in Figure 12, we found that the surface of discharge cavity of the micro-EDM was more uniformed like a grapefruit peel is shown in Figure 12a, while the surface of ECM was smooth and evenly circled as shown in Figure 12b. Desktop micro-ECM is an excellent process for drilling the micro-holes in WC-Co material without leaving burrs. However, it is difficult to control the electrolytic current as well as to improve the control accuracy of the desktop micro-ECM machining and change different machining parameters so that we can enhance the machining performance in terms of material removal rate, average diameter, taper angle and roundness.

### 3.4. Discussion

A low-cost desktop micro-ECM was developed for tungsten cemented carbide micro-hole drilling in this study. The semi-mass production of micro-ECM hole drilling is possible using commercial electrode tools. Using a spindle micro-tool with a screw slot, the desktop micro-ECM is able to perform micro-hole drilling by the micro-ECM process. Moreover, compared with commercial ECM, the desktop micro-ECM has more potential applications in tungsten cemented carbide micro-hole drilling with a very low cost in the future.

## 4. Conclusions

At present, there are few studies focusing on WC-Co micro-hole drilling by micro-ECM. In this paper, a low-cost desktop micro-ECM developed for WC-Co micro-hole drilling machining has been created. The effects of working voltage and electrolytic current on the material removal and surface roughness were investigated. The surface quality was evaluated by optical microscope and SEM observations. Based on the results obtained from this research, some conclusions were found.

The machining parameters and the performance of micro ECM in this paper showed that the ideal values of the working voltage and electrolytic current at 10 V and 3 A, are possible to fabricate good quality micro holes on WC-Co materials. The surface of micro-ECM machining was smoother, while the friction coefficient and wear rate were lower than EDM. WC-Co can be completely removed by the micro-ECM machining of electrochemical dissolution.

In summary, WC-Co micro-hole drilling machining can be achieved by the developed desktop micro-ECM. The experimental results demonstrated that high machining efficiency with good surface quality is achievable for practical applications with the optimal machining parameters.

## Figures and Tables

**Table 1:** Machining parameters of the desktop micro-ECM.

Machining Type	DC-ECM
Workpiece Material	WC-Co
Electrolyte	Sodium hydroxide (NaOH) solution
Concentration	5%
Workpiece Thickness	0.3 mm
Tool Diameter	150 μm
Machining Depth	455 μm
Machining Voltage	5 V, 10 V, 15 V, 20 V

**Table 2:** Data of feeding depth of the desktop micro-ECM.

Current (A)	Time (min)								
	0.00	5.00	10.00	15.00	20.00	25.00	30.00	35.00	40.00
1 (0.5 A)	0	30	80	110	160	210	260	340	455
2 (1 A)	0	60	145	180	245	350	455	-	-
3 (2 A)	0	150	200	300	370	455	-	-	-
4 (3 A)	0	170	255	345	455	-	-	-	-

**Table 3:** Data of micro-tool changes.

Tool (μm)	Voltage (V)			
	5 V	10 V	15 V	20 V
Before Machining	3645	3620	3615	3632
After Machining	3620	3615	3637	3647
Tool Change	−25	−5	12	15

**Table 4:** Data of inlet and outlet diameter of micro-holes.

Diameter (μm)	Times						
	1	2	3	4	5	6	7
Horizontal Inlet Diameter	163	164	164	157	178	161	167
Vertical Inlet Diameter	162	165	158	158	177	178	159
Horizontal Outlet Diameter	154	155	156	121	123	141	128
Vertical Outlet Diameter	153	160	161	122	124	142	130
